# The Transcriptomic Profile of Watermelon Is Affected by Zinc in the Presence of *Fusarium oxysporum* f. sp. *niveum* and *Meloidogyne incognita*

**DOI:** 10.3390/pathogens10070796

**Published:** 2021-06-23

**Authors:** Kasmita Karki, Tim Coolong, Chandrasekar Kousik, Aparna Petkar, Brendon K. Myers, Abolfazl Hajihassani, Mihir Mandal, Bhabesh Dutta

**Affiliations:** 1 Department of Plant Pathology, University of Georgia, Tifton, GA 31793, USA; Kasmita.Karki@uga.edu (K.K.); Aparnap@uga.edu (A.P.); Brendon.Myers25@uga.edu (B.K.M.); abolfazl.hajihassani@uga.edu (A.H.); 2 Department of Horticulture, University of Georgia, Athens, GA 30602, USA; tcoolong@uga.edu; 3 Vegetable Laboratory, USDA, ARS, Charleston, SC 29414, USA; shaker.kousik@usda.gov; 4 Department of Biology, Claflin University, Orangeburg, SC 29115, USA

**Keywords:** watermelon, zinc, disease, *Fusarium*, nematode, plant hormone, transcriptomics, fungi, defense

## Abstract

Zinc (Zn) accumulation and deficiency affect plant response to pests and diseases differently in varying pathosystems. The concentrations of Zn in plants aid in priming defense signaling pathways and help in enhanced structural defenses against plant pathogens. Studies are lacking on how concentrations of Zn in watermelon plants influence defense against two important soil-borne pathogens: *Fusarium oxysporum* f. sp. *niveum* (FON) and southern root-knot nematode (RKN, *Meloidogyne incognita*). In this study a comparative transcriptomics evaluation of watermelon plants in response to high (1.2 ppm) and low (0.2 ppm) levels of Zn were determined. Differential transcript-level responses differed in watermelon plants when infected with FON or RKN or both under high- and low-Zn treatment regimes in a controlled hydroponics system. Higher numbers of differentially expressed genes (DEGs) were observed in high-Zn-treated than in low-Zn-treated non-inoculated plants, in plants inoculated with FON alone and in plants inoculated with RKN alone. However, in the co-inoculated system, low-Zn treatment had higher DEGs as compared to high-Zn treatment. In addition, most DEGs were significantly enriched in hormone signal transduction and MAPK signaling pathway, suggesting an induction of systemic resistance with high-Zn concentrations. Taken together, this study substantially expands transcriptome data resources and suggests a molecular potential framework for watermelon-Zn interaction in FON and RKN.

## 1. Introduction

The Fusarium wilt fungus (*Fusarium oxysporum* f. sp. *niveum*, FON) and the root-knot nematode (*Meloidogyne incognita* (Kofoid and White) Chitwood; RKN) are important soil-borne pathogens causing severe damage in watermelon production throughout the world [1,2,3,4]. FON can infect and induce symptoms in watermelon plants at any growth stage [5]. Symptoms of Fusarium wilt at the seedling stage include seedling dieback and a scorched appearance. In mature plants, tissue chlorosis and unilateral wilting can be observed after loss of turgor pressure. As the disease progresses, complete vine wilting is often observed. FON has four physiological races (0, 1, 2, and 3) based on variability in aggressiveness on differential cultivars [6]. RKN is an obligatory parasite that induces galls on the infected root system of a susceptible host. Galls disrupt the vascular system of plants making them grow poorly and can lead to plant death under heavy infestation [7,8]. Pre-plant fumigant application with methyl bromide (MeBr) was often used and had been effective in reducing both FON and RKN in the watermelon production system. Phasing out of MeBr in accordance with the Montreal Protocol (1993) resulted in limited options for managing both soil-borne pathogens. 

Triploid seedless watermelon cultivars, that are currently cultivated widely, do not possess a high level of resistance against FON race 2 [9]. To further complicate the issue, FON race 3 has also been identified in several states of the U.S. [6]. Proline (prothioconazole; Bayer Crop Science) is the only effective fungicide labeled for use on watermelon to manage FON [10]. Increased selection pressure on FON pathogen populations might lead to increased resistance to the fungicide. Grafting susceptible watermelon scions to interspecific hybrid squash (*Cucurbita maxima* Duch. Ex Lam. × *C. moschata* Duch. Ex Poir) and bottle gourd (*Lagenaria siceraria* (Molina) Standl.) can be a viable option for FON management. Two drawbacks with bottle gourd and *C. moschata* × *C. maxima* hybrid rootstocks are their susceptibility to root-knot nematode and the higher expense of grafted plants [11]. Therefore, available disease control options should be combined with alternative strategies for the integrated management of FON.

Plant disease resistance is affected by the plants’ genetics and by the environment in which it is grown, including nutrient deficiencies and toxicity [12,13,14]. Induced resistance has been proposed as one of the explanations for the interaction between specific nutrients and degree of resistance to plant pathogens [15]. Induced resistance is a plant-based defense system that is elicited by specific environmental stimuli, which enables them to resist biotic challenges [16]. These defense responses include the oxidative burst, changes in cell composition and synthesis of antimicrobial compounds such as phytoalexins [17]. The involvement of mineral nutrients in inducing resistance has been studied in several plant-pathogen systems. For instance, several reports indicate that potassium salts can be used for the induction of host-resistance against powdery mildew on cucumber (*Cucumis sativus*) [18], pepper (*Capsicum annuum*) [19], tomato (*Solanum lycopersicum*) [20] and sugar beet (*Beta vulgaris*) [21]. Potassium application has also been shown to increase downy mildew in muskmelon (*Cucumis melo*), caused by *Pseudoperonospora cubensis*, and Microdochium patch caused by *Microdochium nivale* in creeping bentgrass (*Agrotis stolonifera*) [22,23]. Despite these reports, the impact of a particular nutrient cannot be generalized for all plant-pest/pathogen systems, either globally or individually [12,24]. Therefore, a more detailed understanding of the relationship between plant responses to nutritional status and biotic stresses is essential.

Zinc plays a crucial role in the plant response to pests and diseases. Plants naturally absorb high concentrations of metals such as Zn from the substrate as a self-defense mechanism against pathogens and herbivores [25,26,27]. Metal ions may activate defense reactions through a signaling pathway or by plant fortification. Several studies have demonstrated that Zn fertilization decreases disease severity of Phytophthora root rot (*Phytophthora megasperma*) and common leaf spot (*Pseudopeziza medicaginis*) in alfalfa (*Medicago sativa* L.), peach gummosis (*Botryosphaeria dothidia*) in peach (*Prunus persica*) and early blight (*Alternaria solani*) in potato (*Solanum tuberosum*) [28,29,30]. However, a protective concentration of Zn against certain pathogens can also induce increased susceptibility to another pathogen on the same host [31]. Despite these conflicting reports on Zn and disease resistance and susceptibility, information is lacking on how Zn would affect susceptibility and resistance of watermelon plants against FON and RKN. It is not understood if transcriptome level changes can occur especially in pathways that are related to induced resistance. In the present study, we utilized a hydroponic system to evaluate if varying levels of Zn with or without challenge inoculation with either FON or RKN or both can affect genes in pathways related to induced resistance.

## 2. Results

### 2.1. Overview of the RNA-Seq Results

In order to explore the transcript-level changes in watermelon amended with different levels of Zn and pathogen inoculation (FON or RKN or both), cDNA libraries (*n* = 45) were constructed. Three biological replicates were used for each treatment. A total of 800,231,485 raw reads were generated. After removing the low-quality reads and trimming the adapter sequences, we obtained 787,477,379 (98.41%) clean reads. Using HISAT2 software, 765,227,330 (97.30%) clean reads were mapped to the reference watermelon genome, and 724,923,705 (92.15%) unique reads were obtained. The Q20 percentage was over 98%, and the Q30 percentage was over 98.49% (Table 1).

### 2.2. Differentially Expressed Genes (DEGs)

**Figure 1:**
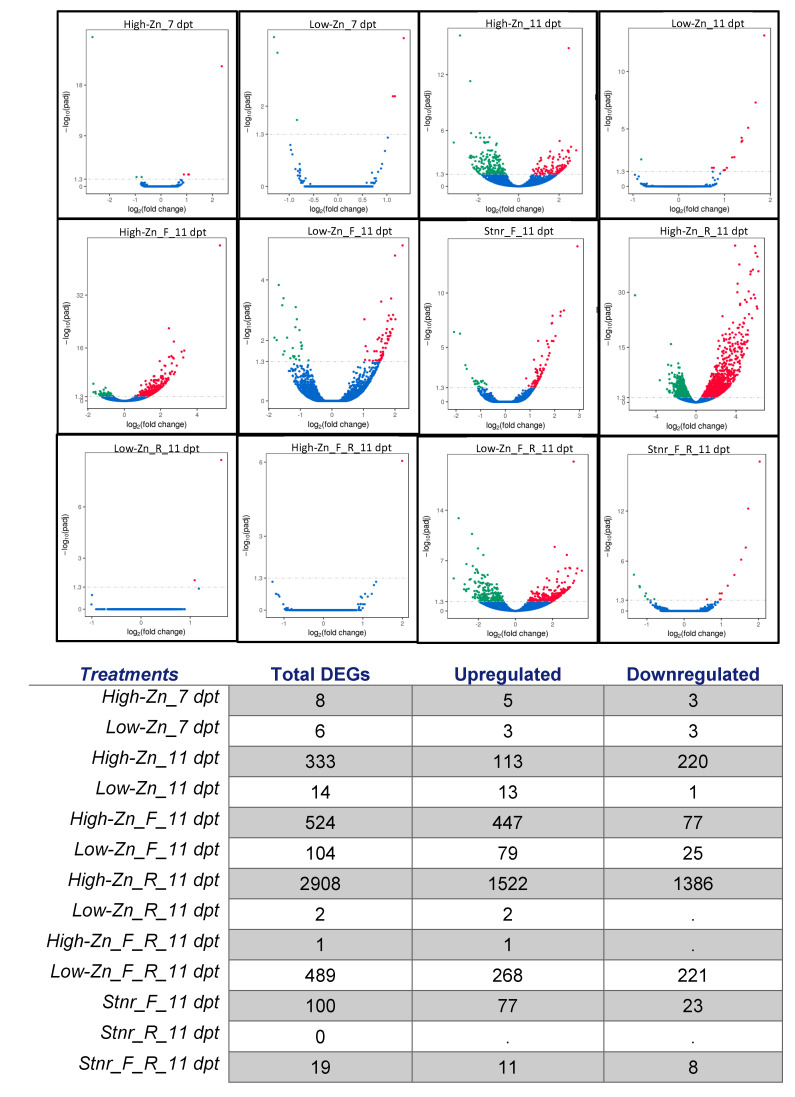
Scatter plot with differentially expressed genes (DEGs) at 7 and 11 days post-treatment (dpt), compared with non-inoculated micronutrient control (Stnr) in high-Zn (HZ) and low-Zn (LZ) treated plants inoculated with *Fusarium oxysporum* f. sp. *niveum* (F) or *Meloidogyne incognita* (R) or both (F_R). Red dot indicates upregulated genes while green dot indicates downregulated genes.

A comparative gene expression analysis of high-Zn (HZ) and low-Zn (LZ) treated plants under inoculated (FON (F) or RKN (R) or both (F_R)) and non-inoculated treatments was conducted. The non-inoculated micronutrient control (Stnr) was used as a control nutrient solution. In non-inoculated plants, the number of DEGs in plants treated with high-Zn was higher than plants treated with low-Zn at both 7 and 11 days post-treatment (dpt) (Supplementary Figure S1 and Figure 1). At 7 dpt, under high-Zn treatment eight DEGs (5 up, 3 dn) were identified whereas in plants treated with low-Zn treatment six DEGs (3 up, 3dn) were obtained. At 11 dpt, there were 333 DEGs (113 up, 220 dn) in plants treated with high-Zn whereas 14 DEGs (13 up, 1dn) in plants treated with low-Zn were identified.

In plants inoculated with FON alone, a similar pattern was observed: the number of DEGs in plants treated with high-Zn (*n =* 524) was higher than Steiner (*n =* 100) and low-Zn (*n =* 104) treated plants. In high-Zn treated plants inoculated with FON, 447 genes were upregulated and 77 genes were downregulated. Steiner treated plants inoculated with FON had 77 upregulated genes and 23 downregulated genes. Low-Zn treated plants inoculated with RKN had 79 upregulated genes and 25 downregulated genes.

Results showed a higher number of DEGs in high-Zn (*n =* 2908) treated plants compared to Steiner (*n =* 0) and low-Zn (*n =* 2) also in plants inoculated solely with RKN. Among the 2908 DEGs in high-Zn treated plants inoculated with RKN, 1522 genes were upregulated, and 1386 genes were downregulated. The two genes in low-Zn treated plants inoculated with RKN were upregulated.

In plants inoculated with both FON and RKN, low-Zn (*n* = 489) treated plants had the highest number of DEGs, followed by Steiner (*n* = 19) and high-Zn (*n* = 1). The one DEG in high-Zn treated plants inoculated with FON and RKN was upregulated. There were 11 upregulated and 8 downregulated genes in Steiner treated plants inoculated with FON and RKN. In low-Zn treated plants inoculated with FON and RKN, 268 genes were upregulated and 221 genes were downregulated.

### 2.3. GO Enrichment Analyses

**Figure 2:**
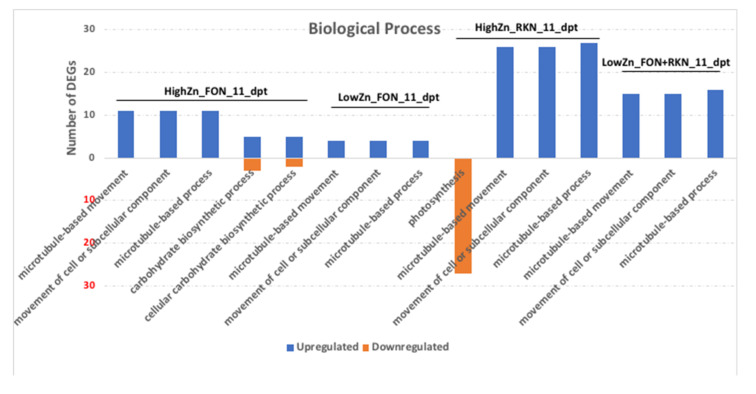
Gene Ontology enrichment analysis of biological process (BP) for upregulated and downregulated genes between high-Zn (HZ) or low-Zn (LZ) treated plants inoculated with *Fusarium oxysporum* f. sp. *niveum* (F) or *Meloidogyne incognita* (R) or both (F_R) and non-inoculated micronutrient control (Stnr) at 11 days post-treatment (dpt) and 3 days post-inoculation.

**Figure 3:**
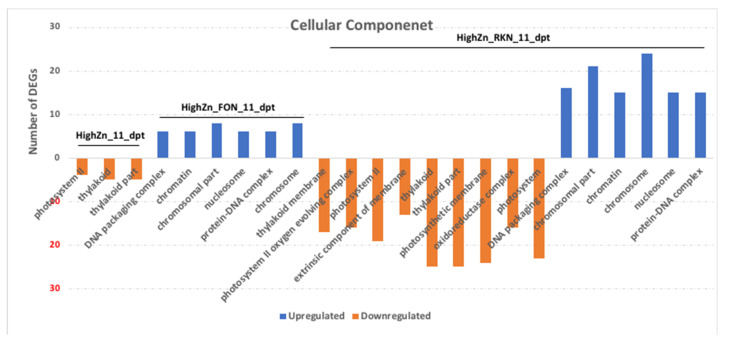
Gene Ontology enrichment analysis of cellular component (CC) for upregulated and downregulated genes between high-Zn (HZ) or low-Zn (LZ) treated plants inoculated with *Fusarium oxysporum* f. sp. *niveum* (F) or *Meloidogyne incognita* (R) or both (F_R) and non-inoculated micronutrient control (Stnr) at 11 days post-treatment (dpt) and 3 days post-inoculation.

GO terms with corrected *p*-value less than 0.05 were considered significantly enriched by differentially expressed genes and are shown in Figure 2 (Supplementary Table S2). Significantly enriched GO terms were observed only at 11 dpt. In the biological process, movement of cell or subcellular component, microtubule-based process and microtubule-based movement were the significantly enriched terms common in high-Zn treated plants inoculated with FON alone, low-Zn treated plants inoculated with FON alone, high-Zn treated plants inoculated with RKN alone, and low-Zn treated plants inoculated with FON and RKN (Figure 2). In the cellular component, thylakoid part, thylakoid and photosystem II were significantly enriched terms in both high-Zn treated plants, with and without inoculation with RKN (alone). In high-Zn treated plants inoculated with FON alone, chromosome, chromosomal part and chromatin were the most enriched terms (Figure 3).

**Figure 4:**
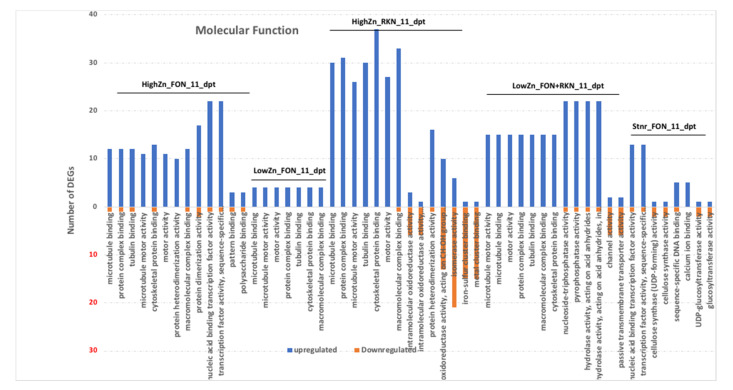
Gene Ontology enrichment analysis of molecular function (MF) for upregulated and downregulated genes between high-Zn (HZ) or low-Zn (LZ) treated plants inoculated with *Fusarium oxysporum* f. sp. *niveum* (F) or *Meloidogyne incognita* (R) or both (F_R) and non-inoculated micronutrient control (Stnr) at 11 days post-treatment (dpt) and 3 days post-inoculation.

Moreover, in molecular function, exopeptidase activity was the only significantly enriched term in low-Zn treated plants (Figure 4). In high-Zn treated plants inoculated with FON alone, transcription factor activity sequence-specific DNA binding, nucleic acid binding transcription factor activity and protein dimerization activity were the most enriched terms. The most enriched term in Steiner treated plant inoculated with FON alone were transcription factor activity sequence-specific DNA binding, nucleic acid binding transcription factor activity and sequence-specific DNA binding. Similarly, tubulin binding, protein complex binding and motor activity were the most enriched terms in low-Zn treated plants inoculated with FON alone. High-Zn treated plants inoculated with RKN alone showed the most enrichment of cytoskeletal protein binding, macromolecular complex binding and protein complex binding. In low-Zn treated plants inoculated with FON and RKN, pyrophosphatase activity, nucleoside-triphosphatase activity and hydrolase activity acting on acid anhydrides in phosphorus-containing anhydrides were the most enriched terms.

### 2.4. KEGG Classification of DEGs

KEGG enrichment results showed that DEGs were classified into multiple signaling pathways (Table 2 and Supplementary Table S3). Significant KEGG (corrected *p*-value < 0.05) enrichment was observed in plants treated with high-Zn at 7 dpt. At 11 dpt plants in treatments: high-Zn inoculated with FON alone, low-Zn inoculated with FON alone, and high-Zn inoculated with RKN alone, showed significant enrichment.

Peroxisome and glycosaminoglycan degradation were significantly enriched with two and one DEGs, respectively, in plants treated with high-Zn at 7 dpt. Two pathways, plant hormone signal transduction and plant-pathogen interaction, were significantly enriched in high-Zn treated plants inoculated with FON alone. In plants treated with low-Zn that were inoculated with FON, alpha-linolenic acid metabolism, valine, leucine and isoleucine biosynthesis and linoleic acid metabolism were significantly enriched. Metabolic pathways, carbon fixation in photosynthetic organisms, photosynthesis, pentose phosphate pathway, starch and sucrose metabolism, ascorbate and aldarate metabolism, carbon metabolism, photosynthesis-antenna proteins and carotenoid biosynthesis were enriched in high-Zn treated plants inoculated with RKN alone.

### 2.5. Investigation of DEGs Associated with Plant Phytohormone Signaling Pathway in Plants Treated with High- and Low-Zn

**Figure 5:**
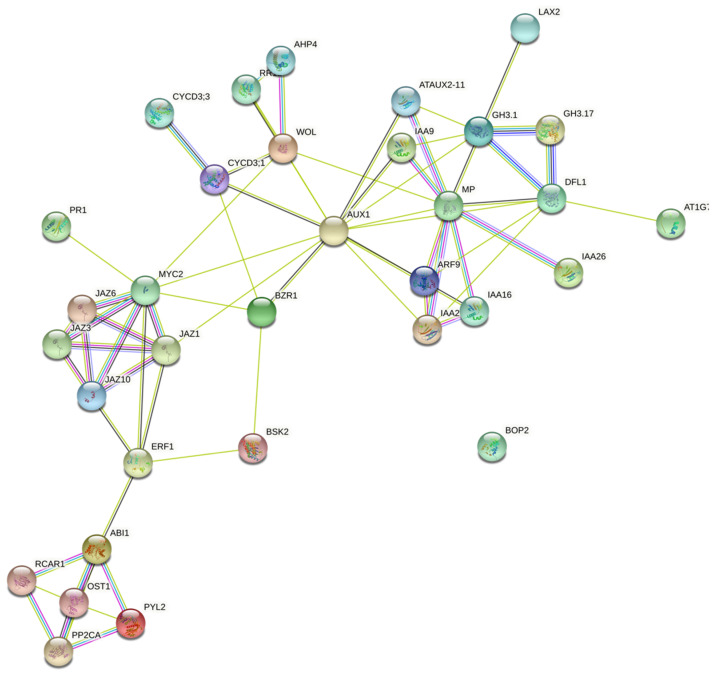
Protein–protein interactome map of hormone related genes affected by high-Zn (HZ) and low-Zn (LZ) in plants inoculated with *Fusarium oxysporum* f. sp. *niveum* (FON), *Meloidogyne incognita* (RKN) or both (FON_RKN), compared to non-inoculated micronutrient control (Stnr) at 11 days post-treatment (dpt) and 3 days post-inoculation.

Phytohormone signaling has been shown to have a crucial regulatory role in plants [32]. Therefore, we have studied the effect of different levels of Zn on changes in the phytohormone signaling pathway. As shown in Table 3, many genes involved in the synthesis and regulation of abscisic acid, auxin, brassinosteroid, cytokinin, ethylene, jasmonic acid and salicylic acid were affected. The data indicate a synergistic regulation of these hormonal related DEGS as shown in Figure 5 through protein–protein interactome map studies using STRING database https://string-db.org/ (accessed on 16 May 2021) with *Arabidopsis thaliana* as the reference organism.

Jasmonic acid (JA) is a major class of plant hormones involved in mediating plant response to biotic and abiotic stress. Four genes predicted to be jasmonate zim domain protein (JAZ: ClCG07G014490, ClCG07G005870, ClCG06G001800, ClCG03G014890) and one gene predicted to be transcription factor MYC2-like protein (MYC2: ClCG10G001590) were induced. Number as well as expression of these genes was higher in high-Zn treatments (high-Zn inoculated with FON and RKN alone) (Table 3).

Salicylic acid (SA) is a key phytohormone that induces plants to produce systemic acquired resistance (SAR) to defend against pathogens [33,34]. Nonexpressor of pathogenesis-related genes 1 (NPR1: ClCG04G004170) and pathogenesis-related protein1 (PR-1: ClCG02G007190) are key regulators in the SA-dependent pathway (Nobuta et al. 2007). In high-Zn treated plants inoculated with RKN alone, one NPR1 gene was significantly upregulated, while one PR-1 gene was significantly downregulated.

Ethylene plays a vital role in plant development and the initiation of defense mechanisms against pathogens [35]. Only one gene associated with the ethylene signaling pathway, ethylene-responsive transcription factor (ERF1/2: ClCG05G004370), was induced in high-Zn treated plants inoculated with RKN alone.

Genes related to abscisic acid, auxin, brassinosteroid and cytokinin signaling pathways were differentially expressed with the difference in Zn level. In the abscisic acid pathway, serine/threonine-protein kinase SRK2 (SnRK2: ClCG00G003890), abscisic acid receptor PYR/PYL family (PYR/PYL: ClCG05G017790, ClCG05G000910), and protein phosphatase 2C (PP2C: ClCG03G001230, ClCG03G016180, ClCG05G008820) were affected. Both the genes related to the abscisic acid pathway were downregulated in plants treated with high-Zn, while in high-Zn treated plants inoculated with RKN, 60% of these genes were upregulated.

In the auxin signaling pathway, SAUR family protein (SAUR: ClCG05G018470), auxin responsive GH3 gene family (GH3: ClCG07G012040, ClCG05G015030, ClCG05G010400, ClCG01G017190), auxin-responsive protein IAA (AUX/IAA: ClCG11G006270, ClCG11G003560, ClCG07G013340, ClCG07G000600, ClCG06G011420, ClCG06G008840, ClCG05G004670, ClCG01G018130), auxin transporter-like protein 3 (AUX1: ClCG02G011740, ClCG02G004210), and auxin response factor (ARF: ClCG09G009470, ClCG05G018490, ClCG07G011440) were affected. A maximum number of genes were differentially regulated in the high-Zn treatments inoculated with FON and RKN alone. In plants treated with high-Zn, one auxin response factor (ClCG09G009470) was upregulated while another one (ClCG05G004670) was downregulated. In high-Zn treated plants inoculated with FON, all six genes (ClCG02G011740, ClCG05G010400, ClCG05G015030, ClCG05G018490, ClCG06G008840, ClCG11G006270) were upregulated. In high-Zn treated plants inoculated with RKN, 12 out of 15 genes were upregulated. Both low-Zn treated plants inoculated with FON and Steiner treated plants inoculated with FON had one upregulated gene. In low-Zn treated plants inoculated with FON and RKN, two (ClCG05G010400, ClCG05G015030) out of four genes were upregulated. In the brassinosteroid pathway, cyclin D3 (CYCD3: ClCG08G016160, ClCG05G020230, ClCG02G006220, ClCG05G010220) and brassinosteroid signaling positive regulator family protein (BZR1/2: ClCG08G015300) were affected, and all of these genes were upregulated. Similarly, histidine phototransfer protein (AHP: ClCG09G000190), Arabidopsis histidine kinase 2/3/4 (cytokinin receptor) (CRE1: ClCG11G001010) and type-B Arabidopsis response regulator (B-ARR: ClCG05G002710) were affected in the cytokinin pathway.

### 2.6. Investigation of DEGs Associated with MAPK and Zinc Finger Proteins Signaling Pathway in Plants Treated with High- and Low-Zn

**Figure 6:**
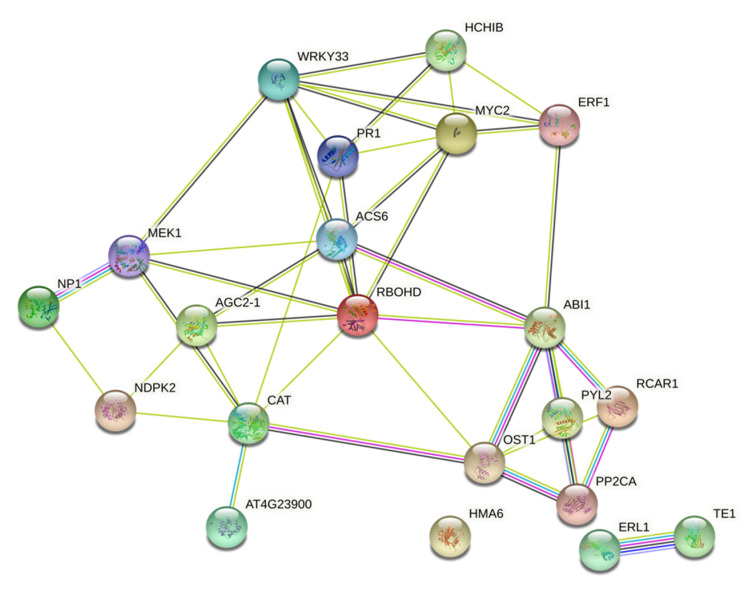
Protein–protein interactome map of genes involved in MAPK signaling affected by high-Zn (HZ) and low-Zn (LZ) in plants inoculated with *Fusarium oxysporum* f. sp. *niveum* (F), *Meloidogyne incognita* (R) or both (F_R), compared to non-inoculated micronutrient control (Stnr) at 11 days post-treatment (dpt) and 3 days post-inoculation.

Previous studies have demonstrated that the MAPK signaling pathway widely exists in eukaryotic organisms, participates in plant growth, and responds to abiotic and biotic stress [36,37]. As shown in Table 4 and Figure 6, a total of 26 genes in MAPK signaling were significantly affected. Results showed that the effect in terms of number and induction level of genes was higher in high-Zn treatments (high-Zn, high-Zn inoculated with FON alone and high-Zn inoculated with RKN alone). Genes such as WRKY DNA-binding protein 33 (WRKY33: ClCG03G000130, ClCG10G022500), respiratory burst oxidase protein D (RbohD: ClCG10G013100), MYC2 (ClCG10G001590, ClCG07G007900) and 1-amicocyclopropane-1-carboxylate synthase 6 (ACS6: ClCG07G007900) were significantly upregulated in high-Zn treated plants inoculated with FON and RKN alone.

In addition, the current study also investigated the regulation of zinc finger proteins (ZFP) in plants treated with high- and low-Zn. A previous report has indicated that plant zinc finger transcription factors are positive regulators of plant immunity, and the association of zinc finger domains in NBS-LRR resistance proteins is well conserved in plants and regulates the plant defense mechanism against diverse plant pathogens [38,39]. As shown in Table S4, a significant change in the regulation of zinc finger proteins in response to high-zinc (80 DEGs) and low-zinc (5 DEGs) treatment (Table S4) was observed. Results showed that 40 genes were upregulated by >two-fold under high-Zn inoculated with RKN alone, 12 genes under high-Zn inoculated with FON alone, and three genes on high-Zn uninoculated treatments. A significant upregulation of ZFPs on high-Zn, as compared to low or normal zinc conditions, supports our hypothesis of priming immunity against RKN and FON by activating the SA mediated signaling with increased transcription of downstream pathogenesis related (PR) genes.

### 2.7. Confirmation of Infection in FON and RKN Inoculation Plants

**Figure 7:**
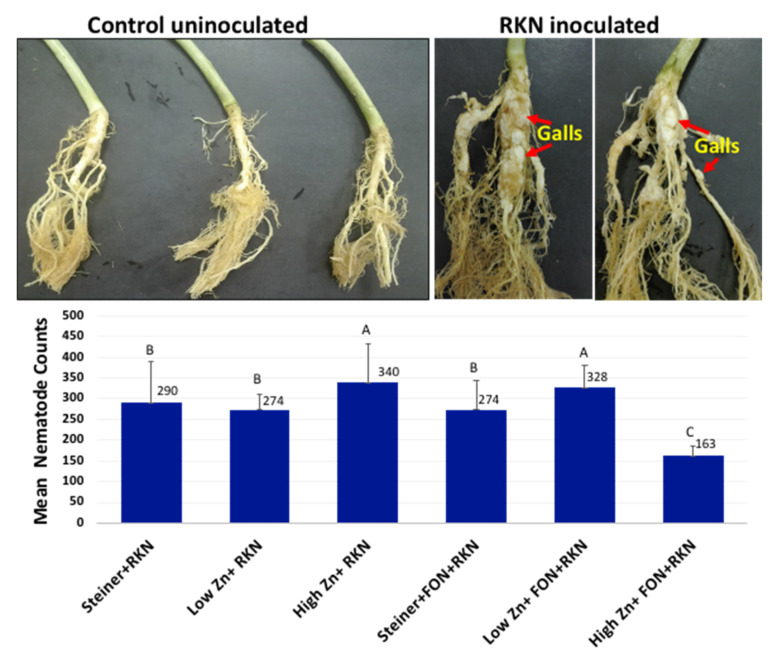
RKN gall formation symptoms and comparative mean nematode counts during RKN disease progression in watermelon roots under high-Zn (HZ) and low-Zn (LZ) conditions in plants inoculated along with *Fusarium oxysporum* f. sp. *niveum* (F) or *Meloidogyne incognita* (R) alone, as compared to non-inoculated control (micronutrient control (Stnr)) at 30dpt. Relative mean nematode counts (egg + J2) were counted in RKN infected samples (three biological replicates). Mean number of RKN adults for each treatment (t) were calculated using Proc Glimmix with Beta distribution for percentage response variable in SAS 9.4 (SAS Institute).

FON was re-isolated from 100% of the plants that were inoculated with FON or co-inoculated with RKN but not from non-inoculated Steiner control or plants only inoculated with RKN. Isolates were tested for FON by conventional PCR as described earlier [40]. One hundred percent of the putatively isolated FON colonies from infected plants were confirmed as FON using the PCR assay. No difference in plants being colonized by FON for all treatments and no Fusarium wilt symptoms were observed. Also, no differences in root gall formation between treatments were observed, except for a decrease in total nematode counts in plants inoculated with both FON and RKN under high zinc treatment (Figure 7). Galls were present only on plants inoculated with RKN or RKN co-inoculated with FON but were absent on non-inoculated or plants only inoculated with FON.

## 3. Discussion

In the present study, a whole transcriptome analysis was performed using RNA-seq in inoculated and non-inoculated (FON, RKN or both) watermelon plants in response to high- and low-Zn levels. We found a higher number of DEGs in high-Zn than in low-Zn-treated plants that were not inoculated; also in plants that were inoculated with FON or RKN alone. However, the effect of high-Zn was not consistent in a co-inoculated system; low-Zn treated plants inoculated with FON and RKN had higher DEGs than high-Zn treated plants inoculated with FON and RKN. These findings indicate that high-Zn treatment can alter differential expression of genes in watermelon, but the level of expression is not consistent across individual (FON or RKN) or co-inoculated (FON and RKN) pathosystems. We identified that the expression level of many genes associated with the phytohormone signaling pathway and the MAPK signaling pathway was affected in high-Zn treated plants, particularly in plants inoculated with FON or RKN alone. As the goal of this study was to evaluate the systemic response of watermelon away from the point of inoculation/infection, leaf tissues instead of root tissues were used for assessments. Within the time frame of the greenhouse study, we did not observe any wilting in plants inoculated with FON, RKN or both. However, fungal isolation and confirmation from the hypocotyl region confirmed the roots were indeed colonized by FON.

Hormone signaling pathways play significant roles in regulating interactions between plants and microorganisms [41,42]. We observed an increased expression of JA pathway genes: JAZ and MYC2 in high-Zn treated plants inoculated with FON or RKN alone. The core signal transduction mechanism of JA signaling comprises JAZ and MYC. Specific JAZ/TFs are generated by JAZ and different TFs that explicitly regulate many downstream responses [43]. The JAZ-MYC module triggers the plant defense response against pathogen infection by increasing the concentration of defense compounds such as indole alkaloids, terpenoid phytoalexins and often through airborne signals (e.g., green leaf volatiles and volatile terpenes) [44]. Gallego et al. [45] also found that surplus Zn can potentiate plant defense responses, especially in the synthesis of JA and it’s signaling pathway, thus improving plant resistance in *Arabidopsis thaliana* against *Alternaria brassicicola*. In addition, we observed an increased expression of NPR1 in high-Zn treated plants inoculated with RKN. NPR1 plays an integral part in the efficacy of the plant defense response. It activates PR gene expression by recruiting TGA transcription factors [46]. *Arabidopsis* NPR1 mutants showed decreased PR gene expression and increased susceptibility to pathogens [47]. Also, the ERF1/2 gene involved in ethylene signaling was upregulated in high-Zn treated plants inoculated with RKN. Activation of ERF genes is known to enhance plant disease resistance [48]. This could mean that high-Zn concentration in watermelon plants modulated induced resistance against FON and RKN. It is further justified by the number of genes involved in synthesis and regulation of SA, JA, abscisic acid, auxin, brassinosteroid, and cytokinin that were significantly affected in high-Zn treated plants inoculated with FON or RKN alone.

Plant MAPKs participate in plant growth, development, and responses to endogenous and environmental cues. Studies have shown that MAPKs could be activated by external sensors for cellular reactions [49]. A study conducted by Bi and Zhou [50] demonstrated that several pathogen-secreted effectors inhibit the MAPK cascade, which confirms the involvement of MAPKs in plant-pathogenic interactions and their role in plant response to pathogen invasion. Our study demonstrated that many genes in the MAPK pathway were significantly affected in high-Zn treated plants inoculated with FON or RKN alone. The genes that were significantly upregulated include WRKY33, RbohD, MYC2, and ACS6. Genes in the WRKY family have been confirmed to perform important regulatory functions to modulate pathogen-triggered cellular responses in a variety of plants, and most WRKY factors participate in the salicylic acid signaling pathway. RbohD is responsible for ROS production, which is involved in regulation of immune function against various pathogens [51,52]. As mentioned earlier, MYC2 a triggers defense response via the JA pathway. Similarly, ACS regulates synthesis of ethylene, which plays a positive role in host resistance against fungal and bacterial pathogens [53,54].

**Figure 8:**
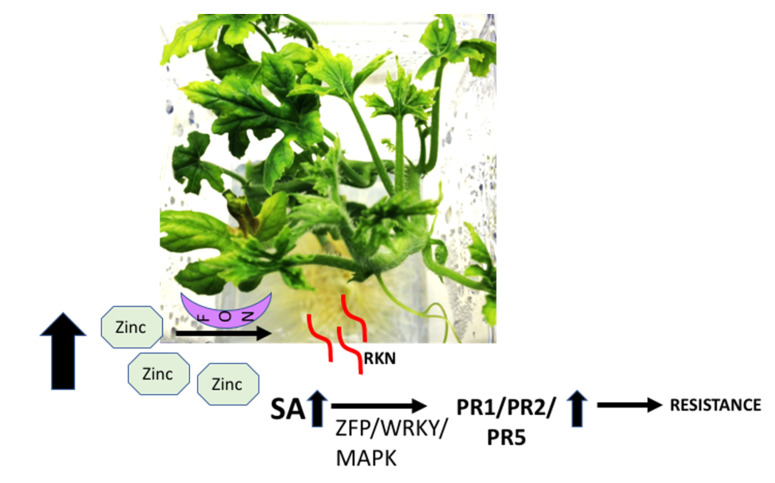
Proposed model of high zinc mediated resistance signaling in watermelon in response to FON and RKN. High level of zinc either in presence of RKN, FON or together induces EDS1 mediated salicylic acid signaling. The increased SA levels further potentiate downstream signaling components (WRKY, zinc finger proteins (ZFP) and map kinase (MAPK)) and trigger nuclear gene expressions of various pathogenesis related (PR1, PR2, PR5) and other defense related genes providing resistance to pathogen.

In summary, we observed that a high-Zn level affects plant–pathogen interaction in watermelon by regulating many crucial plant defense pathways (Figure 8, Figure S1–S6). Multiple genes in several hormone signaling pathways, including SA, JA, ethylene, abscisic acid, auxin, brassinosteroid and cytokinin were significantly affected. However, this effect was limited to FON-only and RKN-only inoculated systems. In the FON and RKN co-inoculated system, the induction of resistance in gall formation was observed; however, changes in DEGs were not observed. It is well known that FON and RKN together can be more severe to watermelon plants and it is possible that such severe interactions might counteract induced host-resistance with altered response in presence of high zinc levels. Pathogens are also known to evolve strategies to counteract Zn-related plant defense [55]. However, plant infection was confirmed at the end of this experiment by the recovery of the fungus and morphological and molecular identification for FON [56] and by presence of galls for RKN in all inoculated plants. Significant differences in terms of disease severity were not observed among inoculated treatments except for the reduced nematode counts when FON and RKN are inoculated simultaneously in high-Zn treated plants compared with Steiner treated plants with both pathogens inoculated. Future research is required to understand the transcriptional and translational basis of this observation. Further study is also required to optimize the level of Zn that can successfully reduce disease severity at the field level based on the effect of Zn ions on natural rhizospheral microbial diversity in natural field conditions. To our knowledge, this is the first study to analyze the effect of Zn level on watermelon in relation to FON or RKN or both infection under controlled hydroponics conditions. 

## 4. Materials and Methods

### 4.1. Experimental Set-Up

Seeds of watermelon cultivar “Sugar Baby”, which is susceptible to both FON and RKN, were sown into sheets of 2.54 cm^2^/cell Rockwool cubes (Grodan Inc., Hedehusene, Denmark) and covered with a thin layer of vermiculite. The sheet was kept moist with water as needed. After germinating, seedlings were watered as needed and fertilized once with a “Steiner solution”, which was modified from the Steiner universal nutrient solution. The composition of the Steiner solution was as follows (mg.L^−1^): N-256 mg.L^−1^ [NH_4_H_2_PO_4_, KNO_3_, Ca(NO_3_)_2_], P-48 mg.L^−1^ (NH_4_H_2_PO_4_), K-304 (KNO_3_), Ca-180 mg.L^−1^ [Ca(NO_3_)_2_], Mg-48 mg.L^−1^ (MgSO_4_), B-1 mg.L^−1^ (H_3_BO_3_), Cu-0.2 mg.L^−1^ (CuSO_4_), Mo-0.1 mg.L^−1^ (Na_2_MoO_4_.2H_2_O), Fe-3 mg.L^−1^ (Fe Chelate; Sequestrene 330), Mn-1 mg.L^−1^ (MnCl_2_) and Zn-0.4 mg.L^−1^ (ZnSO_4_•7H_2_O). Seedlings were maintained for three weeks on Rockwool and then were transferred to plastic containers (43.05 cm W × 30.48 cm D × 19.81 cm H) containing 8L solution of respective micronutrient treatments. The micronutrient treatments were prepared by modifying the Zn composition in the Steiner solution mentioned above. The micronutrient treatments included: high-Zn (HZ: 3X concentration of Zn in Steiner solution, (1.2 ppm)), low-Zn (LZ: 0.5X concentration of Zn in Steiner solution, (0.2 ppm)), and Steiner (Stnr: X concentration of Zn (0.4 ppm)).

Three seedlings along with Rockwool were placed equidistant in a nine-well Styrofoam tray with holes at the bottom so that roots could suspend down and be in contact with the nutrient solution. The Styrofoam tray was then placed over the plastic container filled with micronutrient treatments, as indicated above. Solutions were aerated through a 15.2 cm aquarium air-stone fitted with plastic tubes (0.3-mm diameter) and an air pump (Pentair aquatic eco-systems Inc., Apopka, FL, USA). Plastic containers were maintained to the original volume (8L) by adding dH_2_O every two days. At 8 days post-treatment (dpt), the micronutrient treatments were either inoculated with FON or RKN or co-inoculated with both FON and RKN. For FON inoculation, a 1 mL suspension containing 5 × 10^5^ microconidia/mL (race 2) was applied at the base of the watermelon seedling. For nematode inoculation, a 1ml suspension containing 6000 active RKN J2s (race 3) was added at the base of the seedling. For treatments that were inoculated with both FON and RKN, a similar inoculation approach was adopted and the inocula were applied simultaneously. Plants were maintained at 28 °C mean greenhouse temperature. Three replicates (plastic container with three seedlings each) per treatment were used in the experiment and treatments were arranged in a completely randomized design.

### 4.2. RNA Extraction, Quantification and Integrity Determination

As the goal of this study was to evaluate systemic response of watermelon away from the point of inoculation/infection, leaf tissues instead of root tissues were used for assessments. Leaf samples (*n* = 3 per replicate/treatment) were collected by cutting the 3rd or 4th leaf from the terminal with a pair of sterile scissors at 7 days post-treatment (dpt) and at 11 dpt (3 days post-inoculation (dpi)). Samples were immediately stored in liquid nitrogen and later transferred to a −80 °C freezer at the UGA Tifton Campus laboratory until needed for further analysis. Leaves were ground in liquid nitrogen and total RNA was extracted from 100 mg of ground leaf tissue using the manufacturer’s protocol (RNeasy Plant Mini Kit). Concentrations were determined using a NanoDropTM Lite (Thermo Scientific., Wilmington, DE, USA) and a Qubit^®^ RNA Assay Kit and a Qubit^®^ 2.0 Fluorometer (Life Technologies, Frederick, MD, USA). A total of 45 RNA samples were sent to Novogene Corporation Inc. Sacramento, CA, USA for library construction, sequencing and bioinformatic analysis. The RNA purity was checked using the NanoPhotometer^®^ Spectrophotometer (IMPLEN, CA, USA). RNA integrity was confirmed (RIN > 7) using the RNA Nano 6000 Assay Kit of the Bioanalyzer 2100 system (Agilent Technologies, Santa Clara, CA, USA) with a minimum RNA integrated number of 8.

### 4.3. Library Preparation and Transcriptome Sequencing

A total amount of 1 µg RNA per sample was used to generate RNA-seq libraries using the NEBNext^®^ Ultra^TM^ RNA library Prep Kit for Illimina^®^ (NEB, Ipswich, MA, USA) following the recommendations of the manufacturer, and index codes were added to attribute sequences to each sample. Briefly, mRNA was purified from total RNA utilizing poly-T oligo-attached magnetic beads. Fragmentation was performed using divalent cations under elevated temperature in NEBNext First Strand Synthesis Reaction Buffer (5X). First-strand cDNA was synthesized using random hexamer primer and M- MuLV Reverse Transcriptase (RNAse H). Second strand cDNA synthesis was subsequently performed using DNA Polymerase I and RNase H. Remaining overhangs were converted into blunt ends through exonuclease/polymerase activities. Following the adenylation of 3′ ends of DNA fragments, NEBNext Adaptor with hairpin loop structure was ligated to prepare for hybridization.

In order to select cDNA fragments of preferably 150–200 bp in length, the library fragments were purified using the AMPure XP system (Beckman Coulter, Beverly, MA, USA). Then, 3 µL USER Enzyme (NEB, Ipswich, MA, USA) was used with size-chosen, adapter ligated cDNA at 37 °C for 15 min followed by 5 min at 95 °C before PCR. PCR was then performed with Phusion High-Fidelity DNA polymerase, Universal PCR primers, and Index (X) Primer. Finally, PCR products were purified (AMPure XP system) and the quality of the library was assessed on the Agilent Bioanalyzer 2100 system. The clustering of index-coded samples was performed on the cBOt Cluster Generation System using the P.E. Cluster Kit cBot-H.S. (Illumina) as instructed by the manufacturer. After cluster generation, the preparation of the library was sequenced on the Illumina platform and 150 bp paired-end reads were generated.

### 4.4. Sequence Read Cleanup and Mapping to Genome

First, raw data (raw reads) of the fastq format were processed. Clean reads were obtained by removing reads containing the adapter, reads containing poly-N and low-quality readings (<Q20) from raw data. At the same time, the percentages of reads with Q20, and Q30 were calculated. All downstream analyses were based on clean, high-quality ≥ Q20 data. Reference genome and gene model annotation files were downloaded directly from the genome website (http://cucurbitgenomics.org/pub/cucurbit/genome/watermelon/WCG/v2/) (Accessed on 15 April 2020). Index of the reference genome was developed using hisat2 2.1.0 and paired-end clean reads were aligned to the reference genome Charleston Gray [56,57] using HISAT2.

### 4.5. Quantification of Gene Expression Levels and Differential Expression of Analysis

FeatureCounts v1.5.0-p3 was used to count the read numbers mapped to each gene [55]. Fragments Per kilobase of transcript sequence per million or FPKM of each gene was then calculated based on the length of the gene and reads count mapped to this gene. Differential expression analysis was performed using the DESeq2 R package (1.14.1). The resulting *p* values were adjusted using the Benjamini and Hochberg method for controlling the false discovery rate (FDR). Corrected *p*-value of 0.05 and a log2 (foldchange) of 1 were set as the threshold for significantly differential expression.

### 4.6. GO and KEGG Enrichment Analysis of DEGs

Gene Ontology (GO) enrichment analysis of differentially expressed genes was performed by the clusterProfiler R package, in which gene length bias was corrected. GO terms with corrected *p*-value less than 0.05 were considered to be significantly enriched for a given set of genes. For pathway mapping, the Kyoto Encyclopedia of Genes and Genomics (KEGG) orthology database was adopted (http://www.genome.jp/kegg/) Accessed on 15 April 2020). We used clusterProfiler R package to test the statistical enrichment of differential expression genes in KEGG pathways.

### 4.7. Confirmation of Infection in FON and RKN Inoculation Plants

Visual symptoms of wilting were not observed in plants throughout the experiment. At the end of the experiment, one plant from each container (*n* = 2 containers/treatment), inoculated with FON alone, or co-inoculated with RKN and non-inoculated plants in the Steiner solution, were checked for the presence of FON. Stem pieces (0.5-cm-long) were cut from the base of the main stem of each plant using a pair of scissors sterilized by dipping in 70% ethanol. Stem pieces were surface-disinfested for 1.5 min with 0.6% sodium hypochlorite, rinsed in sterile water and placed on a semi-selective peptone pentachloronitrobenzene agar medium. Plates were incubated at 25 °C for 7 days. Fungal isolates were then microscopically identified based on morphological criteria [58] and further were confirmed as FON by PCR assay with FON specific primers Fon-1/Fon-2 [40]. Percentage of plants infested with FON as determined by morphological and PCR confirmatory assays were recorded. For plants inoculated with RKN, 10 cm of roots closest to the base of plants were sampled and evaluated, as galls were observed only in this portion. Rockwool plugs were removed from the root system, and roots were washed and rated visually for the presence of galls as described earlier [2,4]. Mean number of RKN adults for each treatment (t were calculated using Proc Glimmix with Beta distribution for percentage response variable in SAS 9.4 (SAS Institute). The effects of Zn application and pathogen exposure (FON or RKN or both) on RKN adult numbers in watermelon roots were also analyzed as described above.

## Figures and Tables

**Table 1:** Overview of the watermelon leaf RNA sequencing (RNA-Seq) data.

Sample Name	Raw Reads	Raw Bases	Clean Reads	Total Mapped Reads	Uniquely Mapped Reads	Q20 (%)	Q30 (%)
**HighZn_7 dpt**	5.9 × 10^7^	8.93	5.8 × 10^7^ (98.39%)	5.5 × 10^7^ (97.29%)	5.2 × 10^7^ (92.49%)	98.31	94.70
**LowZn_7 dpt**	5.0 × 10^7^	7.50	4.9 × 10^7^ (98.38%)	4.7 × 10^7^ (97.52%)	4.5 × 10^7^ (92.44%)	98.32	94.77
**Stnr_7 dpt**	4.9 × 10^7^	7.50	4.9 × 10^7^ (98.26%)	4.9 × 10^7^ (97.24%)	4.6 × 10^7^ (91.88%)	98.27	94.67
**HighZn_11 dpt**	5.1 × 10^7^	7.76	5.0 × 10^7^ (98.18%)	4.9 × 10^7^ (97.5%)	4.7 × 10^7^ (93.07%)	98.15	94.34
**HighZn_F_11 dpt**	5.3 × 10^7^	8.10	5.3 × 10^7^ (98.52%)	5.1 × 10^7^ (97.22%)	4.9 × 10^7^ (92.26%)	98.22	94.54
**HighZn_F_R_11 dpt**	5.3 × 10^7^	8.05	5.2 × 10^7^ (98.68%)	5.1 × 10^7^ (96.99%)	4.8 × 10^7^ (91.42%)	98.38	94.94
**HighZn_R_11 dpt**	5.9 × 10^7^	9.00	5.9 × 10^7^ (98.45%)	5.6 × 10^7^ (96.75%)	5.2 × 10^7^ (91.19%)	98.24	94.49
**LowZn_11 dpt**	5.1 × 10^7^	7.70	5.0 × 10^7^ (98.57%)	4.9 × 10^7^ (97.44%)	4.6 × 10^7^ (92.09%)	98.36	94.87
**LowZn_F_11 dpt**	4.9 × 10^7^	7.33	4.8 × 10^7^ (98.53%)	4.6 × 10^7^ (97.53%)	4.4 × 10^7^ (92.83%)	98.23	94.51
**LowZn_F_R_11 dpt**	5.4 × 10^7^	8.20	5.3 × 10^7^ (98.44%)	5.0 × 10^7^ (97.12%)	4.7 × 10^7^ (91.61%)	98.37	94.86
**LowZn_R_11 dpt**	4.9 × 10^7^	7.43	4.8 × 10^7^ (98.24%)	4.9 × 10^7^ (97.64%)	4.6 × 10^7^ (92.97%)	98.32	94.74
**Stnr_11 dpt**	5.3 × 10^7^	7.96	5.2 × 10^7^ (98.53%)	5.0 × 10^7^ (97.17%)	4.7 × 10^7^ (91.37%)	98.00	93.96
**Stnr_F_11 dpt**	4.9 × 10^7^	7.47	4.8 × 10^7^ (98.25%)	4.5 × 10^7^ (97.69%)	4.3 × 10^7^ (92.41%)	98.24	94.58
**Stnr_F_R_11 dpt**	5.1 × 10^7^	7.67	5.0 × 10^7^ (98.33%)	6.8 × 10^7^ (97.49%)	6.5 × 10^7^ (93.26%)	98.36	94.84
**Stnr_R_11 dpt**	6.3 × 10^7^	9.50	6.2 × 10^7^ (98.31%)	4.4 × 10^7^ (96.95%)	4.1 × 10^7^ (90.92%)	98.32	94.76

%Q20: The percentage of bases with phred quality score of 20 or higher; %Q30: The percentage of bases with phred quality score of 30 or higher.

**Table 2:** Pathways and gene count of major changes in KEGG enrichment in DEGs.

KEGGID	Description	*p* Value	Count
**High-Zn_7 dpt**			
csv04146	Peroxisome	0.002165	2
csv00531	Glycosaminoglycan degradation	0.01651	1
**High-Zn_FON_11 dpt**			
csv04075	Plant hormone signal transduction	1.41 × 10^−5^	14
csv04626	Plant-pathogen interaction	0.0007	9
**Low-Zn_FON_11 dpt**			
csv00592	alpha-Linolenic acid metabolism	0.00213	3
csv00290	Valine, leucine and isoleucine biosynthesis	0.00363	2
csv00591	Linoleic acid metabolism	0.00417	2
**High-Zn_RKN_11 dpt**			
csv00710	Carbon fixation in photosynthetic organisms	1.73 × 10^−7^	25
csv00195	Photosynthesis	1.58 × 10^−6^	21
csv00030	Pentose phosphate pathway	0.00015	17
csv00500	Starch and sucrose metabolism	0.00021	31
csv00053	Ascorbate and aldarate metabolism	0.00052	15
csv01200	Carbon metabolism	0.00138	48
csv00196	Photosynthesis—antenna proteins	0.00246	8
csv00906	Carotenoid biosynthesis	0.00327	11

**Table 3:** Expression profile of hormone related DEGs.

Treatment	Gene ID	Description	Log2Fold Change	Hormone	Name
HighZn_11 dpt	ClCG00G003890	Kinase family protein	−1.7089	Abscissic acid	SnRK2/OST1
	ClCG01G001770	Unknown protein	0.86607		
	ClCG05G004670	Auxin-responsive protein	−1.02	Auxin	AUX/IAA/ATAUX2-11
	ClCG05G010220	Cyclin D3-1	1.9249	Brassinosteroid	CYCD3/CYCD3
	ClCG05G017790	Abscisic acid receptor PYR1	−1.8846	Abscissic acid	PYR/PYL
	ClCG09G000190	Histidine-containing phosphotransfer protein, putative	−1.9525	Cytokinine	AHP/AHP4
	ClCG09G009470	Auxin response factor	1.5998	Auxin	ARF
HighZn_FON_11 dpt	ClCG02G011740	Auxin transporter-like protein 3	1.2816	Auxin	AUX1
	ClCG03G014890	Jasmonate-zim-domain protein 1	0.84193	Jasmonic acid	JAZ/JAZ1
	ClCG05G010220	Cyclin D3-1	1.8242	Brassinosteroid	CYCD3/CYCD3
	ClCG05G010400	GH3	1.7049	Auxin	GH3
	ClCG05G015030	GH3	1.4005	Auxin	GH3
	ClCG05G018490	Auxin response factor, putative	1.1285	Auxin	ARF
	ClCG05G020230	Cyclin D	1.6576	Brassinosteroid	CYCD3
	ClCG06G001800	jasmonate-zim-domain protein 10 LENGTH = 197	1.1178	Jasmonic acid	JAZ
	ClCG06G008840	Auxin-responsive protein	1.1682	Auxin	AUX/IAA
	ClCG07G014490	Jasmonate ZIM-domain protein 3b	2.033	Jasmonic acid	JAZ
	ClCG08G015300	BES1/BZR1 protein	1.1164	Brassinosteroid	BZR1/2
	ClCG10G001590	Transcription factor MYC2-like protein	2.4574	Jasmonic acid	MYC2
	ClCG11G001010	Histidine kinase 2	−0.87669	Cytokinine	CRE1/WOL
	ClCG11G006270	Auxin transporter-like protein	1.0301	Auxin	AUX/IAA/AUX1
HighZn_RKN_11 dpt	ClCG01G001770	Unknown protein	1.132		
	ClCG01G017190	GH3-1	2.6134	Auxin	GH3/GH3.1
	ClCG01G018130	Auxin-responsive family protein	1.2014	Auxin	AUX/IAA/IAA9
	ClCG02G004210	Auxin transporter-like protein 3	2.2592	Auxin	AUX1/LAX2
	ClCG02G006220	Cyclin D3.2 protein	1.7357	Brassinosteroid	CYCD3
	ClCG02G007190	Cysteine-rich venom protein	−1.3058	Salicylic acid	PR-1
	ClCG02G011740	Auxin transporter-like protein 3	2.6958	Auxin	AUX1/LAX2
	ClCG03G001230	Protein phosphatase 2C	1.0321	Abscissic acid	PP2C/PP2CA
	ClCG03G014890	Jasmonate-zim-domain protein 1	1.8871	Jasmonic acid	JAZ/JAZ1
	ClCG03G016180	Protein phosphatase 2c, putative	1.5072	Abscissic acid	PP2C/ABI1
	ClCG04G004170	BTB/POZ ankyrin repeat protein	2.8402	Salicylic acid	NPR1/BOP2
	ClCG05G000910	Abscisic acid receptor PYR1	−0.78244	Abscissic acid	PYR/PYL/RCAR1
	ClCG05G002710	Kinase family protein	0.99557	Cytokinine	B-ARR/BSK2
	ClCG05G004370	Ethylene-responsive transcription factor 1B, putative	1.9806	Ethylene	ERF1/2
	ClCG05G008820	Protein phosphatase 2c, putative	2.2817	Abscissic acid	PP2C/PP2CA
	ClCG05G010220	Cyclin D3-1	3.8147	Brassinosteroid	CYCD3/CYCD3
	ClCG05G010400	GH3	2.8392	Auxin	GH3/GH3.1
	ClCG05G015030	GH3	2.6677	Auxin	GH3
	ClCG05G017790	Abscisic acid receptor PYR1	−1.8103	Abscissic acid	PYR/PYL2
	ClCG05G018470	SAUR-like auxin-responsive protein family LENGTH = 154	2.753	Auxin	SAUR
	ClCG05G018490	Auxin response factor, putative	2.8171	Auxin	ARF/MP
	ClCG05G020230	Cyclin D	3.1068	Brassinosteroid	CYCD3
	ClCG06G001800	jasmonate-zim-domain protein 10 LENGTH = 197	1.1222	Jasmonic acid	JAZ/JAZ10
	ClCG06G008840	Auxin-responsive protein	1.7638	Auxin	AUX/IAA/IAA27
	ClCG06G011420	Indole-3-acetic acid inducible 29	−1.2596	Auxin	AUX/IAA
	ClCG07G000600	Auxin-responsive protein	−1.535	Auxin	AUX/IAA/IAA16
	ClCG07G005870	Jasmonate ZIM-domain protein 2	1.7448	Jasmonic acid	JAZ/JAZ3
	ClCG07G011440	auxin response factor 15 LENGTH = 598	3.0858	Auxin	ARF/ARF9
	ClCG07G013340	Auxin-responsive protein	1.671	Auxin	AUX/IAA/IAA26
	ClCG07G014490	Jasmonate ZIM-domain protein 3b	3.8539	Jasmonic acid	JAZ/JAZ6
	ClCG08G015300	BES1/BZR1 protein	1.2391	Brassinosteroid	BZR1/2
	ClCG08G016160	Cyclin D3.1 protein	0.77641	Brassinosteroid	CYCD3
	ClCG09G009470	Auxin response factor	2.1743	Auxin	ARF/ARF9
	ClCG10G001590	Transcription factor MYC2-like protein	3.1504	Jasmonic acid	MYC2
	ClCG11G003560	response regulator 12 LENGTH = 596	−1.018	Auxin	AUX/IAA/RR12
LowZn_FON_11 dpt	ClCG05G015030	GH3	1.389	Auxin	GH3
	ClCG07G014490	Jasmonate ZIM-domain protein 3b	1.9189	Jasmonic acid	JAZ
	ClCG07G005870	Jasmonate ZIM-domain protein 2	1.5802	Jasmonic acid	JAZ
	ClCG10G001590	Transcription factor MYC2-like protein	1.7156	Jasmonic acid	MYC2
LowZn_FON_RKN_11 dpt	ClCG01G001770	Unknown protein	1.2498		
	ClCG05G004670	Auxin-responsive protein	−1.4502	Auxin	AUX/IAA/ATAUX2-11
	ClCG05G010220	Cyclin D3-1	2.3229	Brassinosteroid	CYCD3/CYCD3
	ClCG05G010400	GH3	1.4416	Auxin	GH3
	ClCG05G015030	GH3	1.9852	Auxin	GH3
	ClCG05G020230	Cyclin D	2.6506	Brassinosteroid	CYCD3
	ClCG07G005870	Jasmonate ZIM-domain protein 2	1.7035	Jasmonic acid	JAZ
	ClCG07G012040	Auxin-responsive GH3 family protein	−1.1949	Auxin	/GH3.17
	ClCG10G001590	Transcription factor MYC2-like protein	1.8151	Jasmonic acid	MYC2
SteineRKN_FON_11 dpt	ClCG05G010400	GH3	1.2947	Auxin	GH3
	ClCG07G014490	Jasmonate ZIM-domain protein 3b	2.2331	Jasmonic acid	JAZ
	ClCG03G014890	Jasmonate-zim-domain protein 1	1.285	Jasmonic acid	JAZ/JAZ1
	ClCG10G001590	Transcription factor MYC2-like protein	1.8784	Jasmonic acid	MYC2
LZ_FON_11 dpt	ClCG05G015030	GH3	1.389	Auxin	GH3/DFL1

**Table 4:** Expression profile of MAPK signaling DEGs.

Treatment	Gene_id	Description	Log2 Fold Change	Name
HighZn_11 dpt	ClCG00G003890	Kinase family protein	−1.7089	SnRK2
	ClCG05G017790	Abscisic acid receptor PYR1	−1.8846	PYR/PYL
	ClCG07G011590	Nucleoside diphosphate kinase	−1.1574	NDPK2
	ClCG09G008560	Kinase family protein	−1.0076	OXI1
	ClCG11G001650	Protein kinase	1.2952	ANP1
HighZn_FON_11 dpt	ClCG03G000130	WRKY transcription factor 07	1.5388	WRKY33
	ClCG07G007900	1-aminocyclopropane-1-carboxylate synthase	1.7681	ACS6
	ClCG10G001590	Transcription factor MYC2-like protein	2.4574	MYC2
	ClCG10G013100	Respiratory burst oxidase-like protein	1.5519	RbohD
	ClCG10G022500	WRKY 7 transcription factor	2.0214	WRKY33
HighZn_RKN_11 dpt	ClCG01G020770	Chitinase	−1.0037	ChiB
	ClCG02G007190	Cysteine-rich venom protein	−1.3058	PR1
	ClCG02G015820	Protein kinase, putative	0.98462	ANP1
	ClCG03G000130	WRKY transcription factor 07	2.0512	WRKY33
	ClCG03G001230	Protein phosphatase 2C	1.0321	PP2C
	ClCG03G003540	Nucleoside diphosphate kinase	−1.1075	NDPK2
	ClCG03G016180	Protein phosphatase 2c, putative	1.5072	PP2C
	ClCG05G000910	Abscisic acid receptor PYR1	−0.78244	PYR/PYL
	ClCG05G004370	Ethylene-responsive transcription factor 1B, putative	1.9806	ERF1
	ClCG05G008820	Protein phosphatase 2c, putative	2.2817	PP2C
	ClCG05G015780	Leucine-rich receptor-like protein kinase family protein LENGTH = 976	2.7165	ER/ERLs
	ClCG05G017790	Abscisic acid receptor PYR1	−1.8103	PYR/PYL
	ClCG07G007900	1-aminocyclopropane-1-carboxylate synthase	2.5606	ACS6
	ClCG09G013300	Receptor-like protein kinase	2.4712	ER/ERLs
	ClCG09G015420	Copper-transporting atpase paa1, putative	−1.1722	RAN1
	ClCG10G001590	Transcription factor MYC2-like protein	3.1504	MYC2
	ClCG10G013100	Respiratory burst oxidase-like protein	2.6512	RbohD
	ClCG10G022500	WRKY 7 transcription factor	2.1625	WRKY33
	ClCG11G018720	Catalase	−1.4567	CAT1
LZ_FON_11 dpt	ClCG10G001590	Transcription factor MYC2-like protein	1.7156	MYC2
LZ_FON_RKN_11 dpt	ClCG07G011590	Nucleoside diphosphate kinase	−0.96588	NDPK2
	ClCG10G001590	Transcription factor MYC2-like protein	1.8151	MYC2
	ClCG11G001650	Protein kinase	1.5157	ANP1
Stnr_FON	ClCG03G000130	WRKY transcription factor 07	1.0872	NDPK2
	ClCG07G007900	1-aminocyclopropane-1-carboxylate synthase	1.2221	MYC2
	ClCG10G001590	Transcription factor MYC2-like protein	1.8784	ANP1
	ClCG10G013100	Respiratory burst oxidase-like protein	1.3053	RbohD
	ClCG10G022500	WRKY 7 transcription factor	2.0338	WRKY33

## Data Availability

The transcriptomic sequencing data generated and analyzed in this study is available on request from bhabesh@uga.edu.

## Supplementary Materials

The following are available online at https://www.mdpi.com/article/10.3390/pathogens10070796/s1, Figure S1. Upregulated DEGs associated with diverse Biological Process BP under Hz_RKN condition. Figure S2. Upregulated DEGs associated with diverse Molecular Functions MF under Hz_RKN condition. Figure S3. Upregulated DEGs associated with diverse Cellular Component CC under Hz_RKN condition. Figure S4. Downregulated DEGs associated with diverse Biological Process BP under Hz_RKN condition. Figure S5. Downregulated DEGs associated with diverse Molecular Functions MF under Hz_RKN condition. Figure S6. Downregulated DEGs associated with diverse Cellular Component CC under Hz_RKN condition. Table S1. List of DEGs in different treatments. Table S2. Differentially expressed genes related to Gene Ontology enrichment analysis at 11 dpt. Table S3. KEGG analysis of differentially expressed genes at 11 dpt. Table S4. DEGs associated with zinc finger protein(s) and pathogenesis related (PR) proteins in response to high and low Zn treatment.
