# Supplementary figures and images for: The Transcriptomic Profile of Watermelon Is Affected by Zinc in the Presence of Fusarium oxysporum f. sp. niveum and Meloidogyne incognita
Source: Pathogens. 2021 Jun 23;10(7):796. doi: 10.3390/pathogens10070796 (PMC8308719; doi:10.3390/pathogens10070796)

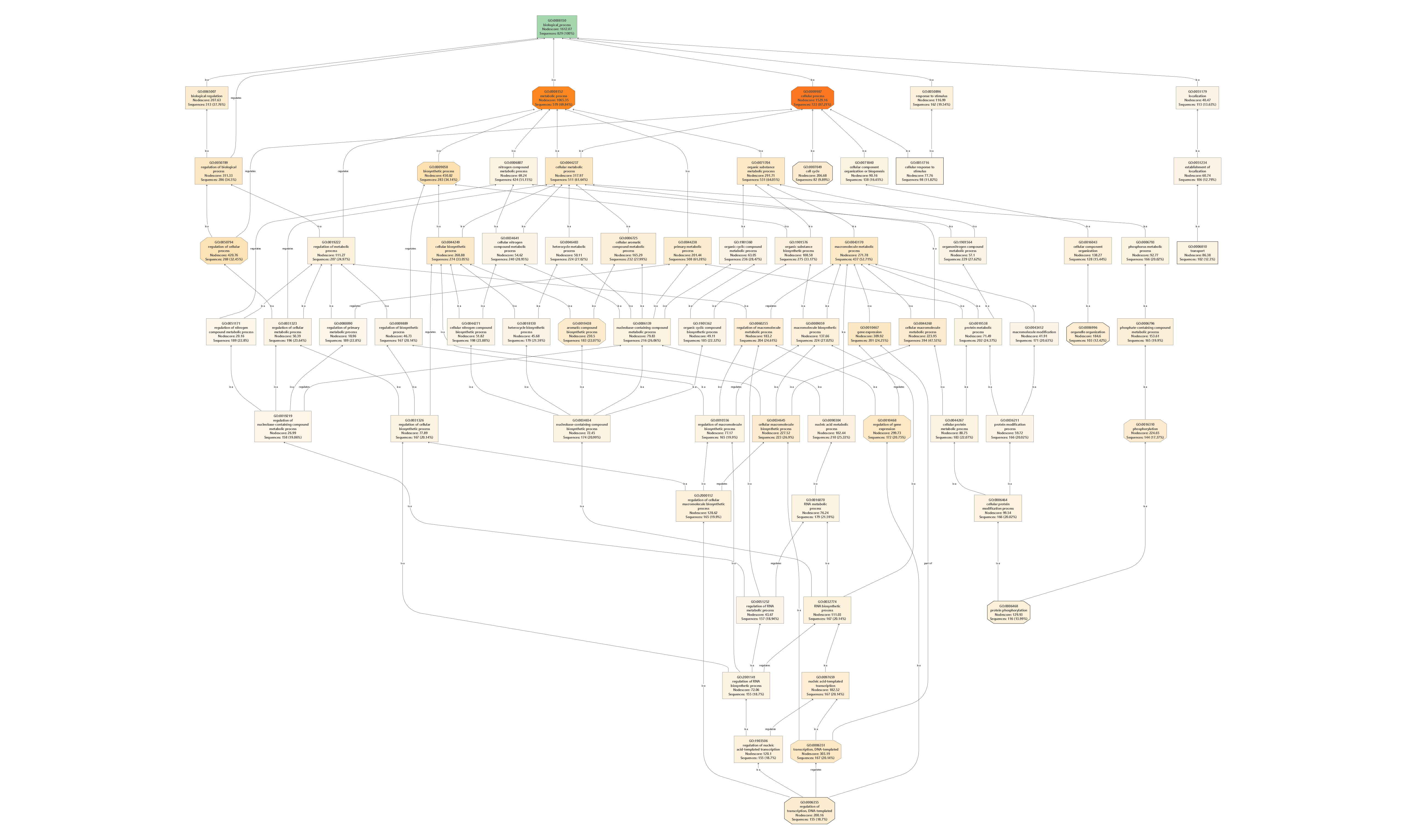

Supplement: Supplementary file 1 [file pathogens-10-00796-s001.zip › Figure S1_omicsbox_graph_BP_Hz_R_upregulated DEGs.png]

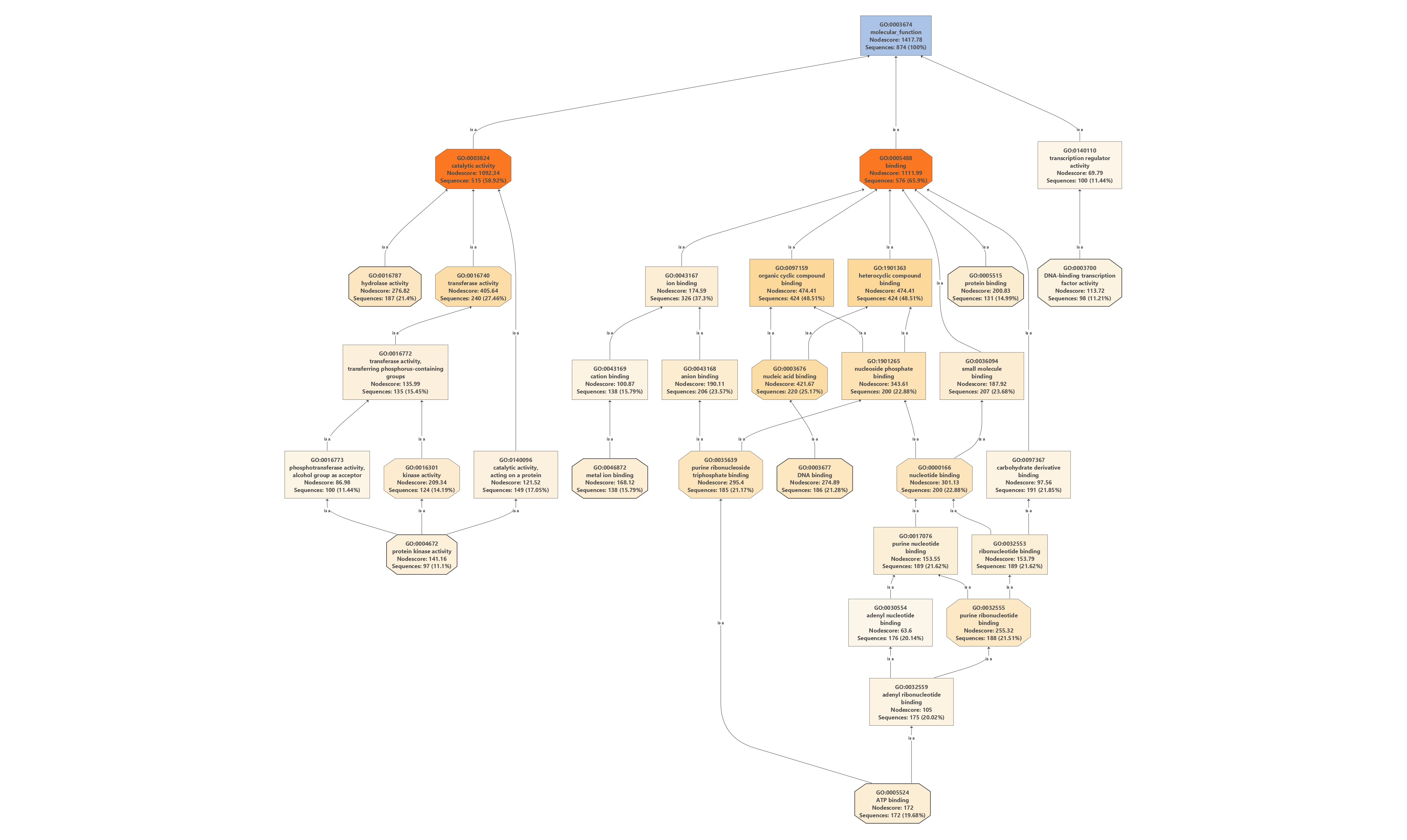

Supplement: Supplementary file 1 [file pathogens-10-00796-s001.zip › Figure S2_omicsbox_graph__MF_Hz_R_upregulated DEGs.png]

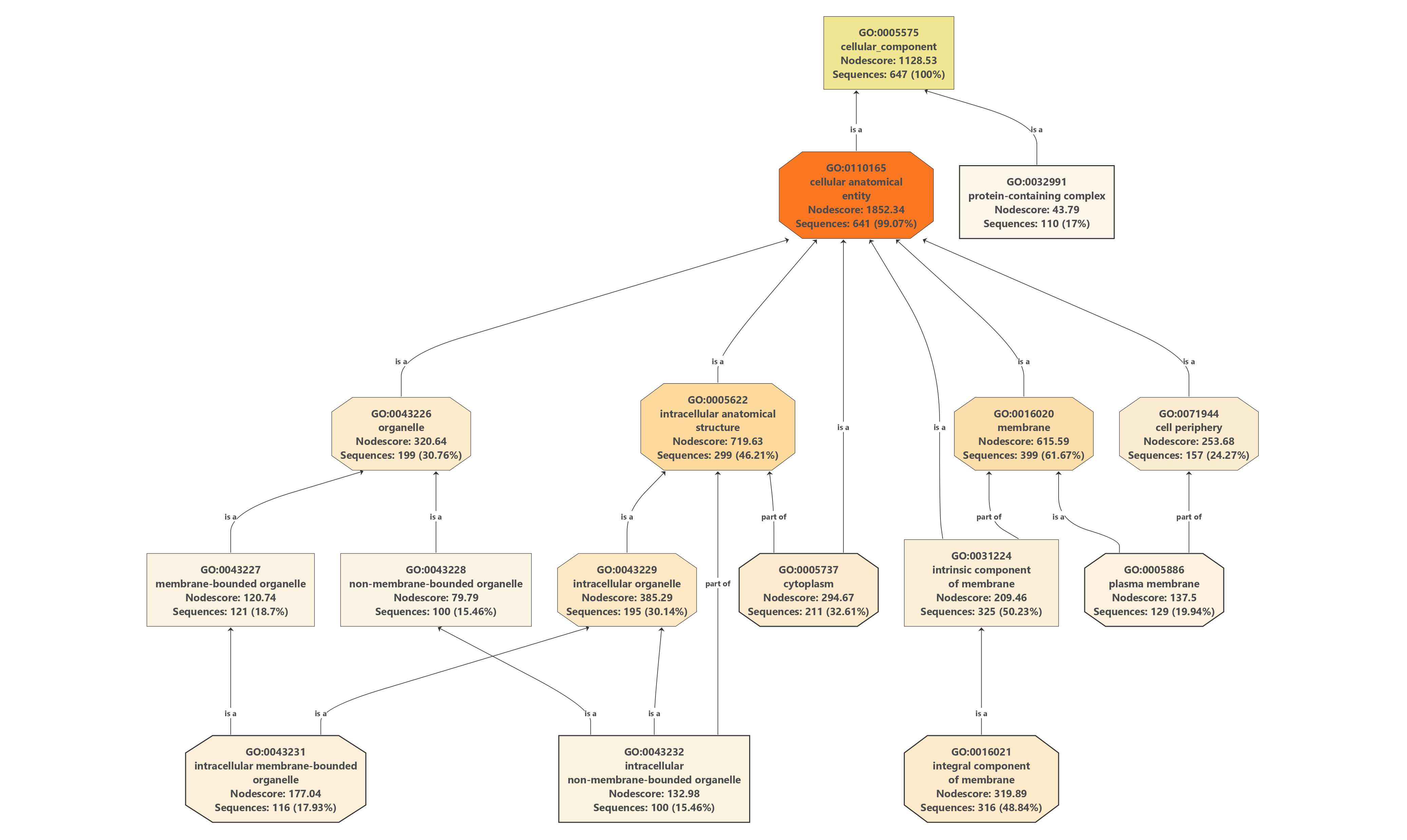

Supplement: Supplementary file 1 [file pathogens-10-00796-s001.zip › Figure S3_omicsbox_graph__CC_Hz_R_upregulated DEGs.png]

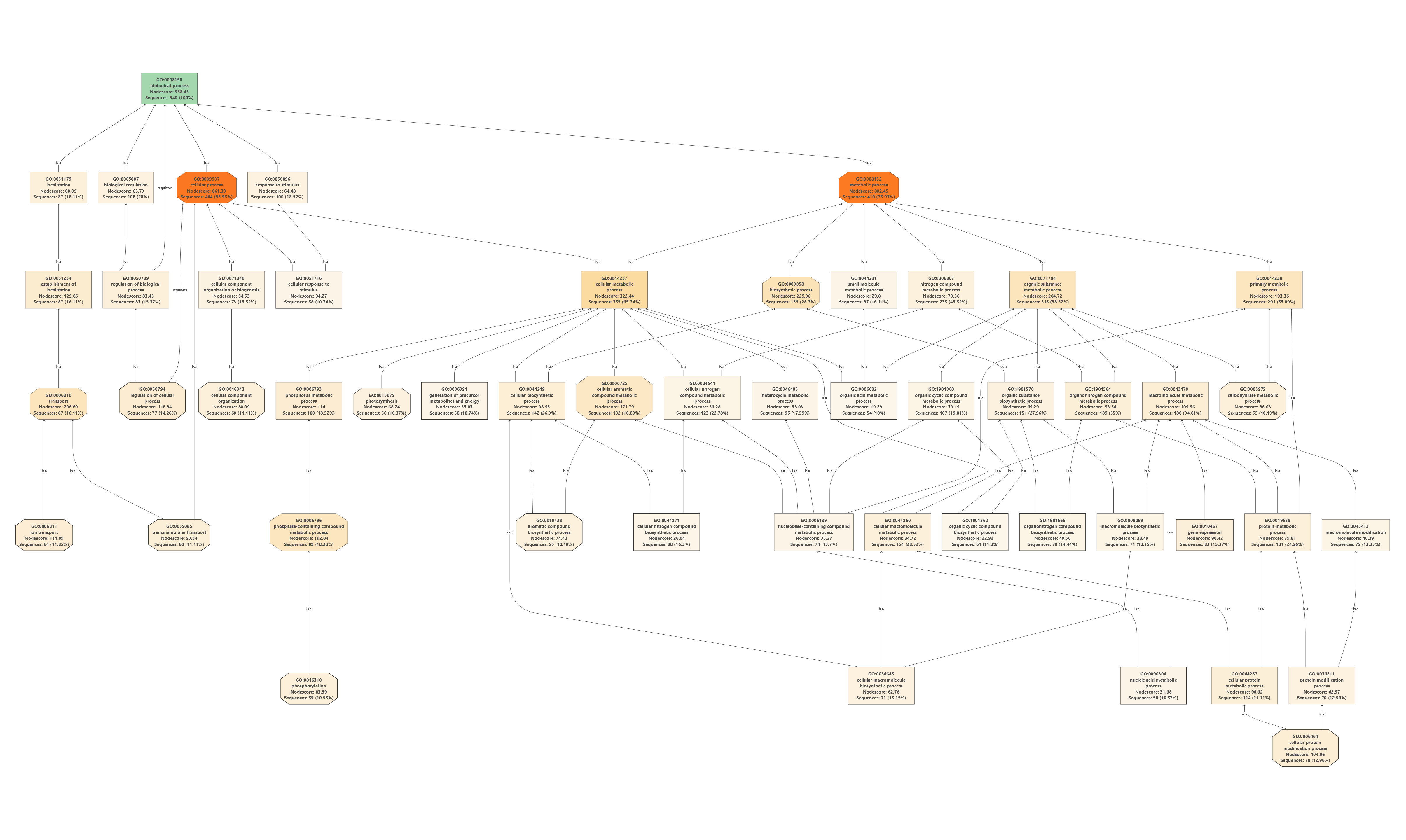

Supplement: Supplementary file 1 [file pathogens-10-00796-s001.zip › Figure S4_omicsbox_graph__BP_Hz_R_downregulated DEGs.png]

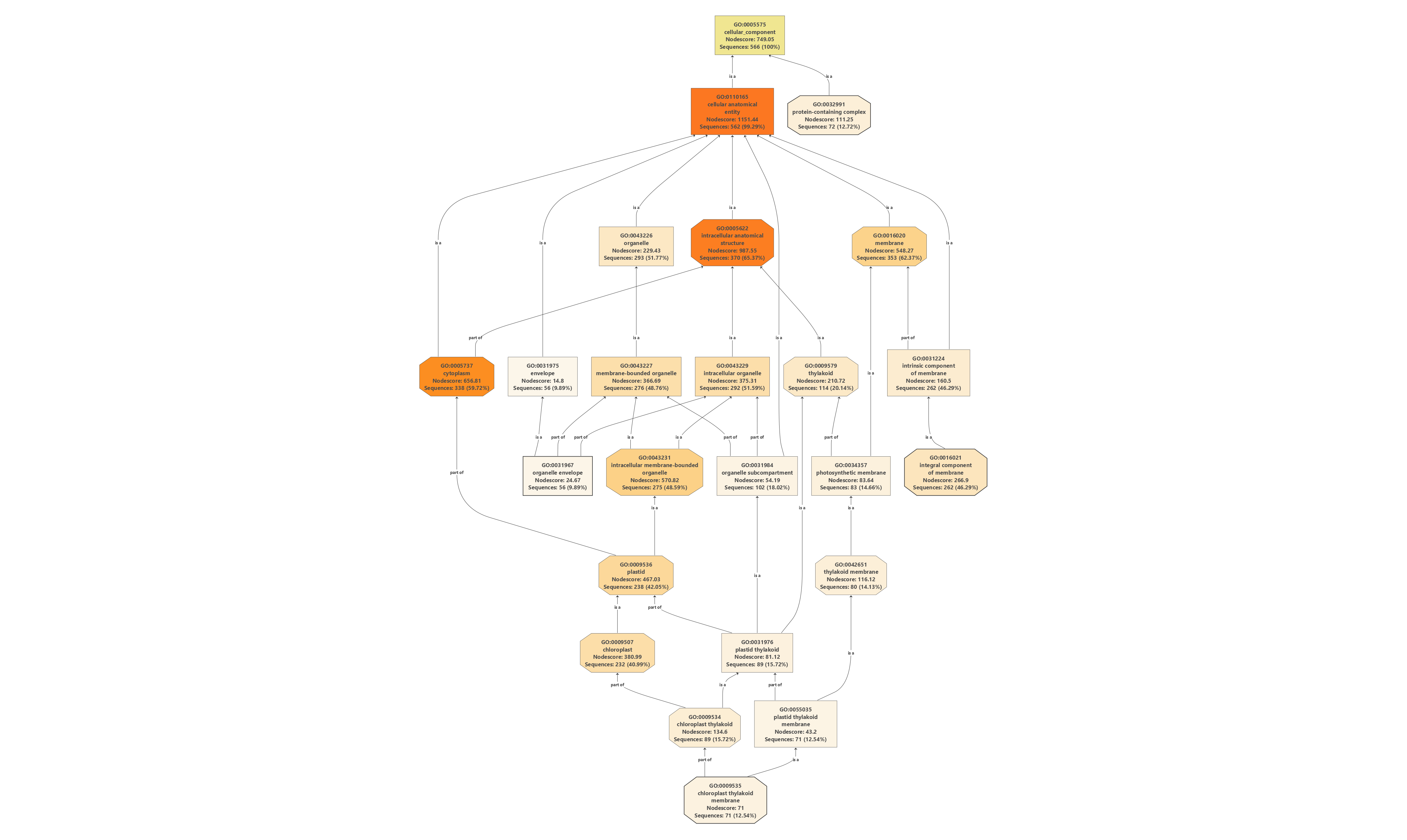

Supplement: Supplementary file 1 [file pathogens-10-00796-s001.zip › Figure S5_omicsbox_graph__MF_Hz_R_downregulated DEGs.png]

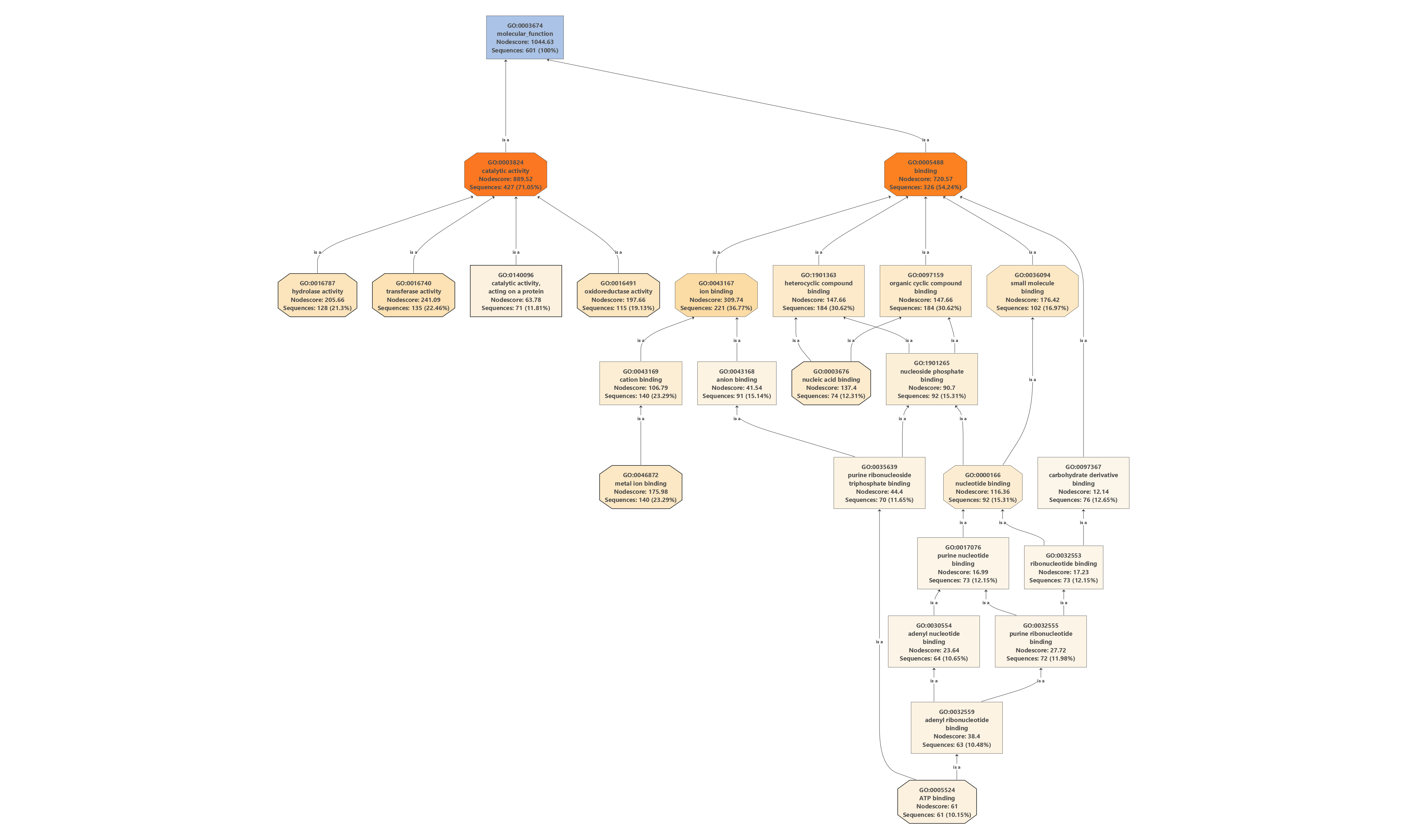

Supplement: Supplementary file 1 [file pathogens-10-00796-s001.zip › Figure S6_omicsbox_graph__CC_Hz_R_downregulated DEGs.png]
